# Effects of Agronomic Practices on Volatile Composition of *Hyssopus officinalis* L. Essential Oils 

**DOI:** 10.3390/molecules16054131

**Published:** 2011-05-19

**Authors:** Armando Moro, Amaya Zalacain, Jorge Hurtado de Mendoza, Manuel Carmona

**Affiliations:** 1 Cátedra de Química Agrícola, E.T.S.I. Agrónomos de Albacete, Universidad de Castilla La Mancha, E- 02071 Albacete, Spain; 2 Sailab, Parc tecnológic del Vallès, Argenters, 5 Ed. I. Bajos D, 08290 Cerdanyola del Vallès, Spain; 3 Albacete Science & Technology Park Foundation, Universidad de Castilla La Mancha, E-02071 Albacete, Spain

**Keywords:** *Hyssopus officinalis*, essential oil, GC-MS, volatile composition, irrigation

## Abstract

The chemical composition of *Hyssopus officinalis* (Lamiaceae) essential oil grown in southeastern Spain was analyzed by GC-MS. Due to the high relevance of this species in the world market, the study is focused on chemical heterogeneity of different oil batches and their extraction yield, cultivated under irrigation and non-irrigation conditions and with different harvesting dates. All essential oil samples have two main terpene compounds which are pinocamphone and *iso*-pinocamphone, accounting for approximately 35–40% of the total oil content. Other relevant compounds were identified, with *β*-pinene, which accounted for 10–17% contribution to the total composition, standing out. Significant differences between their volatile composition have been observed between treatments, being limonene, (E)-*β*-ocimene, pinocarveol, *α*-pinene and *β*-phellandrene the compounds that contributed most to the discrimination. It was also observed that the irrigation period is the most favourable for the cultivation of hyssop in this region, specially for batch 7 which gives the highest extraction yield and the best EO quality.

## 1. Introduction

Like other aromatic plants from the Lamiaceae family, hyssop (*Hyssopus officinalis* L.) has remarkable botanical and commercial interest [1,2]. Due to its great floristic and phytogenic richness, the Iberian Peninsula has the richest spontaneous aromatic plant flora in Europe. This situation has allowed for the establishment of an important production and export industry based on the harvest of wild aromatic plants, which have some problems as a consequence of the great heterogeneity among the chemical composition of the final products and lack of quality control, as it is the case of hyssop extracts.

The extraction from the aerial part (leaves and flowering top) of *H. officinalis* results in an essential oil (EO) with a wide number of applications. It is used mainly for flavouring and food preservation [2,3,4] and for phytotherapeutic uses [5]. 

The chemical heterogeneity of this EO maybe associated to factors such as the different varieties (*ssp. aristatus, montanus, angustifolius, canescens*, etc), chemotype/phenotypes (linalool-rich, etc) and different populations, that are not easily controlled. Some agrotechnical factors, such as fertilization, water supply, and harvesting can be followed to achieve a successful production [1,6,7,8,9]. At the same time, the chemical composition for hyssop oil is now controlled worldwide by the ISO 9841 [10], where 13 compounds are recognized as standards, being pinocamphone, *iso*-pinocamphone and *β*-pinene the most abundant (40–90%).

Due to the high demand for *H. officinalis* L. essential oil on world markets, our study is centred on how the irrigation and non-irrigation treatments over the same cultivation affect the quality of the EO chemical composition, in order to be able to provide some guidance for optimum cultivation conditions to commercial growers.

## 2. Results and Discussion

**Figure 1:**
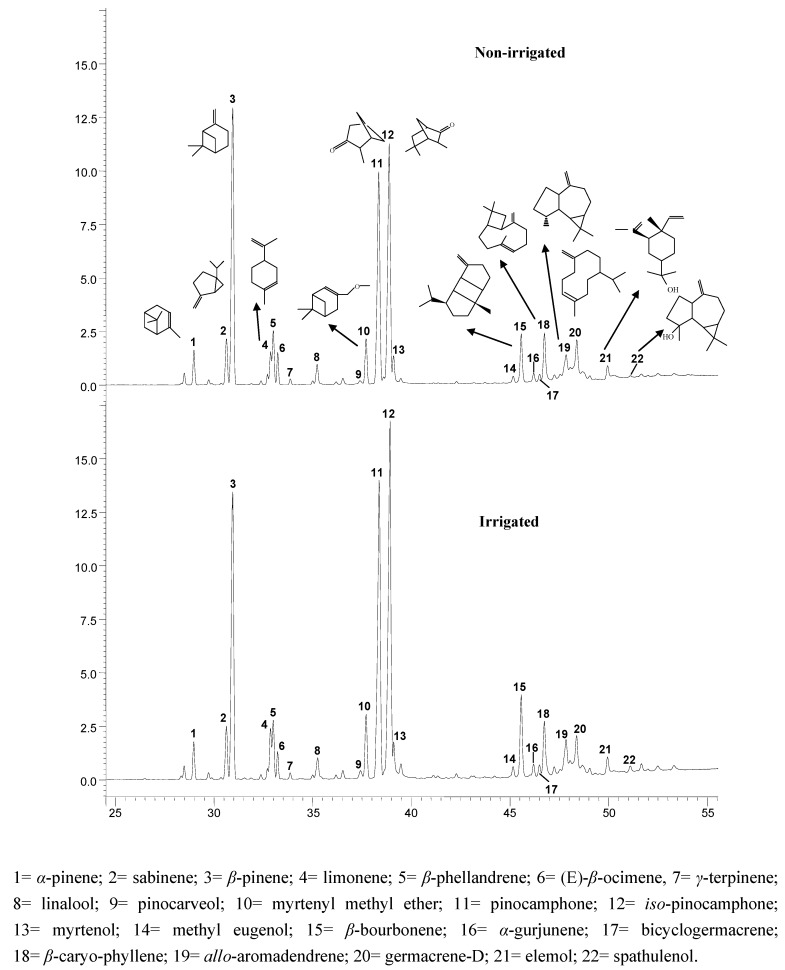
Representative GC profiles of *Hyssopus officinalis* L. from irrigated and non-irrigated crop. Chemical structures for ISO 9841 compounds are shown.

Although *Hyssopus officinalis* L. is a type of crop that needs a low water supply and its cultivation in the Mediterranean area is favourable, a significant water deficit in this type of aromatic crops, may cause negative changes in terms of oil quality [11]. In this regard, the region of Castilla-La Mancha (Albacete) is characterized by semiarid climatic conditions, with a low annual rainfall (300–400 mm) and particularly rigorous Spring seasons (max. value in 150 mm) in terms of drought periods [12]. For each plot (irrigated and non-irrigated) harvesting was carried out during five days close to the full bloom period as there were no choices to harvest the whole plot at once. Each harvest was processed separately in order to study by first time the evolution of the EO extraction yield and quality due to the irrigation effect over an annual crop of hyssop. According to the extraction yield, the lower percentages were obtained for batches 1 and 2, which belong to the non-irrigated samples, whereas the highest significant yield was observed for batch 7 (irrigated conditions). No significant differences were observed among the other conditions assays (batches 3 to 6, and 8 to 10). In terms of EO volatile composition, all batches showed similar volatile profiles described by 22 compounds analysed by GC-MS (Figure 1). 

Thirteen of them are listed in the ISO 9841 [10] normative which is used for certifying EO quality in the international trade (Figure 1). Looking in detail at the EO composition for all samples (Table 1), there are four compounds that are out of range for the values fixed by ISO 9841:2007, *α*-pinene (**1**), *iso*-pinocamphone (**12**), *β*-bourbonene (**15**) and *β*-caryophyllene (**18**). With less frequency, myrtenyl methyl ether (**10**) is out of range in all the irrigated samples (batches 6 to 10) and one non-irrigated (batch 5). Also, *β*-pinene (**3**) is out of range in two non-irrigated sampling (batches 1 and 3), whereas *allo*-aromadendrene (**19**) and germacrene D (**20**) are out of range in only two out of ten samples, batches 7 and 1, respectively.

**Table 1:** Extraction yield (%) and volatile composition (% area) obtained for each type of studied conditions (non-irrigated and irrigated crop) in essential oil of *H. officinalis* L.

			Batch from non-irrigated crop (%)					Batch from irrigated crop (%)					ISO 9841:2007 (E)
			1	2	3	4	5	6	7	8	9	10	
	**Average Oil volume (L/Batch)**		5.25	5.25	6.50	6.63	6.67	6.75	7.00	6.20	6.25	6.25
	**EO yield extraction (%)**		0.35a	0.35a	0.43b	0.44b	0.44b	0.45b	0.47c	0.41b	0.42b	0.42b
	**Compounds identification**	**KI**											%
**1**	*α* -pinene	939	1.87e	1.83de	1.84e	1.77cd	1.75c	1.60ab	1.54a	1.58ab	1.62b	1.61ab	0.4 - 1.5
**2**	Sabinene	975	2.85b	2.75ab	2.74ab	2.73ab	2.70a	2.65a	2.68a	2.64a	2.72a	2.62a	1.0 - 3.5
**3**	*β* -pinene	979	20.33cd	19.88cd	20.54d	19.67cd	19.25bc	18.33ab	17.72a	17.62a	17.73a	17.65a	7.0 - 20
**4**	Limonene	1029	1.73c	1.61b	1.53a	1.57ab	1.58ab	2.09e	2.01e	1.93d	2.03e	2.22f	0.6 - 4.0
**11**	Pinocamphone	1162	17.16a	17.06a	16.87a	17.38ab	17.62ab	18.53c	17.07a	17.94bc	19.33d	19.75d	8.0 - 25
**12**	*iso* -pinocamphone	1175	19.79a	19.36a	19.47a	19.97a	20.62ab	21.43b	24.58c	24.22c	23.65c	23.93c	25 - 45
**10**	myrtenyl methyl ether	1372	2.86a	2.81a	3.01b	3.07b	3.13b	3.15b	3.31c	3.33c	3.33c	3.36c	0.9 - 3.0
**15**	*β* -bourbonene	1388	3.48a	3.91b	4.07bc	4.08bc	4.32c	4.11bc	3.91b	4.05bc	4.19c	4.25c	0.8 - 2.6
**18**	*β* -caryophyllene	1419	3.88c	4.16d	4.16d	3.94cd	3.96cd	3.56b	3.11a	3.29a	3.27a	3.28a	1.0 - 3.0
**19**	*allo* -aromadendrene	1460	2.62a	3.16a	3.07a	2.98a	2.94a	3.03a	3.12a	2.77a	2.58a	2.56a	1.0 - 3.0
**20**	germacrene-D	1481	4.76f	4.43ef	4.28e	4.04de	3.63bcd	3.70cd	3.38abc	3.38abc	3.23ab	3.06a	1.2 - 4.5
**21**	Elemol	1549	0.97a	1.19bc	1.29c	1.19bc	1.23c	0.96a	1.02ab	0.96a	0.91a	0.83a	0.2 -2.5
**22**	Spathulenol	1578	0.14a	0.25ab	0.27ab	0.42b	0.31ab	0.37b	0.30ab	0.32ab	0.34ab	0.29ab	0.1 -1.5
	**Percentage for ISO compounds**		**82.44**	**82.40**	**83.14**	**82.81**	**83.05**	**83.49**	**83.78**	**84.04**	**84.94**	**85.42**	
**5**	*β* -phellandrene	1025	3.14e	3.02de	2.96cd	2.89bcd	2.87abc	2.89bcd	3.92g	3.44f	2.80ab	2.74a	N.I
**6**	(E)- *β* -ocimene	1050	1.57f	1.46e	1.32d	1.31d	1.18c	1.12b	1.15bc	1.09b	1.09ab	1.04a	N.I
**7**	*γ* -terpinene	1059	0.31a	0.28a	0.27a	0.27a	0.27a	0.29a	0.29a	0.29a	0.25a	0.40a	N.I
**8**	Linalool	1096	1.30a	1.23a	1.25a	1.23a	1.28a	1.23a	1.23a	1.13a	1.21a	1.14a	N.I
**9**	Pinocarveol	1184	0.25a	0.25a	0.30b	0.30b	0.34c	0.41d	0.42d	0.46e	0.48f	0.50f	N.I
**13**	Myrtenol	1195	1.82c	1.77bc	1.72bc	1.78c	1.75bc	1.81c	0.69a	1.07ab	1.07ab	1.00a	N.I
**14**	methyl eugenol	1402	0.43a	0.48a	0.45a	0.44a	0.49a	0.47a	0.44a	0.46a	0.48a	0.48a	N.I
**16**	*α* -gurjunene	1409	0.54ab	0.60bc	0.60bc	0.60bc	0.61c	0.58abc	0.55abc	0.53ab	0.48a	0.48a	N.I
**17**	Bicyclogermacrene	1500	0.50a	0.56b	0.56b	0.57b	0.57b	0.53ab	0.49a	0.52ab	0.53ab	0.53ab	N.I
	**Percentage for other compounds (no ISO)**		**9.87**	**9.63**	**9.44**	**9.39**	**9.35**	**9.33**	**9.18**	**9.00**	**8.38**	**8.30**	
	**Percentage of total area compounds**		**92.31**	**92.04**	**92.57**	**92.20**	**92.40**	**92.82**	**92.96**	**93.03**	**93.32**	**93.72**	

*Note:* Different upper script letters between columns indicate significant differences at p > 0.05; N.I.: Not Included in ISO 9841:2007.

The main compounds of hyssop are the terpene ketones pinocamphone (**11**) and *iso*-pinocamphone (**12**), contributing approximately 36 to 41% of the total extract, which have to be taken into account due to their toxicity problems [13]. Both compounds display similar behaviour respect to the harvesting date, as there are no significative (*P* ≤ 0.05) differences between the first five days of harvesting (batches 1 to 5) and from that moment their content increases till the last harvesting date, batch 10 (irrigated conditions) (Table 1). This effect is important for *iso*-pinocamphone (**12**) getting closer to the ISO minimum limit at the end of the period. Similar behaviour was observed for other three compounds, two of them of relevant contribution and included in ISO 9841: myrtenyl methyl ether (**10**) was steadily increasing and *β*-bourbonene (**15**) which increases more rapidly, during the last period. Both of them fall outside the limits established by the ISO normative, as plant maturation increases, the opposite behaviour than that seen for *iso*-pinocamphone (**12**). Finally, pinocarveol (**9**) present in a lesser amount, experienced a sharp increase in its values from batch 1 to batch 10.

Another compound with an important contribution to the volatile composition (17 to 20%) is *β*-pinene (**3**), recognised as one of the most bioactive component of some essential oils and a common constituent of oils from the Lamiaceae family [14,15,16]. In this case, the highest content is observed during the first four harvesting dates (batches 1 to 4), and later it is reduced to a practically stable level over time. Another six compounds share this behaviour decreasing during harvesting time: *α*-pinene (**1**), *β*-caryophyllene (**18**), germacrene D (**20**), *β*-phellandrene (**5**), (E)-*β*-ocimene (**6**) and myrtenol (**13**). In the case of *α*-pinene (**1**), *β*-pinene (**3**), *β*-caryophyllene (**18**) and germacrene D (**20**), their decrement is positive since their content are then included or closer to the ISO limits. 

Finally there is a third group corresponding to the compounds maintaining their content through the whole period or having an erratic performance: sabinene (**2**), *γ*-terpinene (**7**), linalool (**8**), methyl eugenol (**14**), *α*-gurjenene (**16**), bicyclogermacrene (**17**), *allo*-aromadendrene (**19**), elemol (**21**) and spathulenol (**22**).

**Figure 2:**
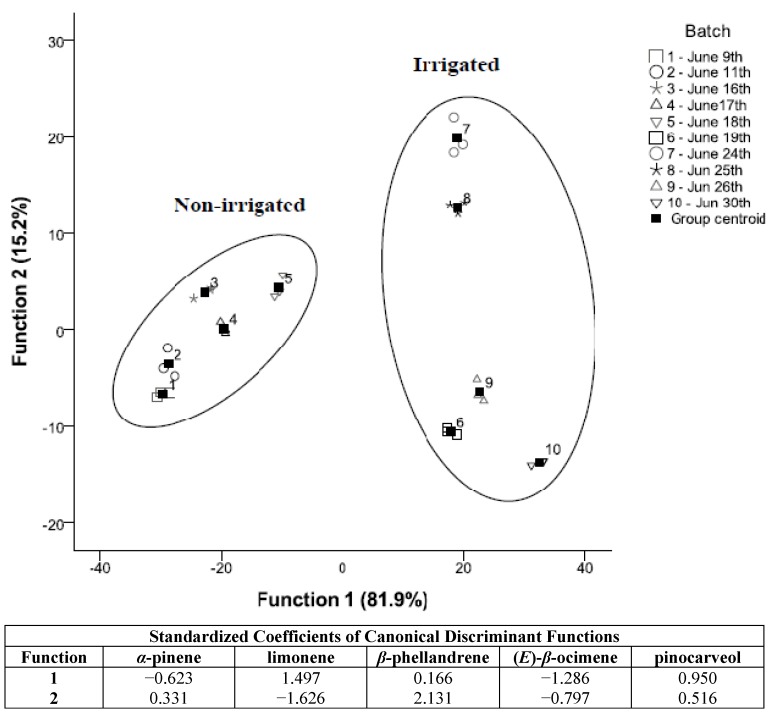
Application of discriminant analysis to the data expressed as data harvest of different samples.

The descriptive analysis presented above was corroborated by a canonical discriminant analysis, carried out using all quantified compounds in order to evaluate if different irrigation and non-irrigation conditions affect the essential oil composition of hyssop EO. Differences were found between treatments (Figure 2), and a total variance of 97.1% was explained by two canonical functions (81.9% and 15.2%). 

The volatile compounds that contributed most to the discriminant model were limonene (**4**), (E)-*β*-ocimene (**6**), pinocarveol (**9**), *α*-pinene (**1**) and *β*-phellandrene (**5**). Limonene (**4**) has a segmented and non-progressive behaviour (Table 1), as its content seems to be more related with the irrigation conditions, and is for this reason useful for discrimination analysis. It is interesting to find out that sample discrimination is due to another three compounds [(*E*)-*β*-ocimene, pinocarveol and *β*-phellandrene] that do not belong to the ISO standard. While (*E*)-*β*-ocimene (**6**), *β*-phellandrene (**5**) and *α*-pinene (**1**) decrease with the harvesting date, only pinocarveol (**9**) increases.

When irrigated and non-irrigated samplings were compared, although there are values out of the range, as mentioned before, the values for *α*-pinene (**1**), *iso*-pinocamphone (**12**) and *β*-caryophyllene (**18**) are closer to the ISO values within non irrigated samples (batches 1 to 5) than irrigated ones (batches 6 to 10). The opposite effect has been observed with *β*-bourbonene (**15**). The EO compositional development of these compounds highlights the irrigation period as the most favourable, and especially when harvesting was carried out during 24 and 25^th^ of June (batches 7 and 8, respectively) (Figure 2), although no significance differences have been observed with the next sampling for most compounds (Table 1).

## 3. Experimental

### 3.1. Plant Material

*Hyssopus officinalis* L. was cultivated and collected on *Dehesa de los Llanos* State, in the province of Albacete (Castilla-La Mancha, Spain), during 2009. Two groups of samples were collected, the ones that were collected from a non-irrigated agriculture plot (20 ha) and the ones collected from an irrigated plot (22 ha). Plant material from each crop type was harvested and distilled on five different days depending on the flowering states of each crop. Non-irrigated samples were collected June the 9^th^, 11^th^, 16^th^, 17^th^, and 18^th^ (batches 1 to 5) and irrigated samples were collected on June 19^th^, 24^th^, 25^th^, 26^th^, and 30^th ^ (batches 6 to 10). All samples were dehydrated at room temperature until they reached a constant weight.

### 3.2. Essential Oil Extraction

The fresh aerial material (stems with their corresponding inflorescence) was distilled in the same state. Extractions of the samples have been carried out on an industrial scale during 1.5 h in three vessels of 5 m^3^ each. Temperature and pressure conditions in the process were 108 °C and 1.1 kg/cm^2^, Respectively. The biomass employed in the distillation was 1,500 kg per batch giving an oil volume, according to the process for each distillation cycle (Table 1). Samples were kept under dark conditions at room temperature till analysis. As a result of this distillation, 10 different batches of hyssop essential oil were obtained corresponding to non-irrigated (batches 1–5) and irrigated (batches 6–10) conditions.

### 3.3. Gas Chromatography-Mass Spectrometry Analysis (GC-MS)

Samples (0.2 µL) were subjected to analysis by capillary gas chromatography. A Varian CP-3800 gas chromatograph (GC) (Palo Alto, CA, USA), equipped with a Saturn 2200 ion trap mass spectrometry (MS) and an CombiPal autosampler (Palo Alto, CA, USA) provided with an Elite-Volatiles Specialty phase capillary column (30 m × 0.25 mm i.d.; 1.4 μm film thickness; Perkin Elmer, Shelton, CT, USA) were used. The chromatographic program was set at 35 °C (held for 10 min), raised to 240 °C at 5 °C/min (held for 30 min); injector temperature, 230 °C; detector temperature, 250 °C; Helium carrier gas flow was 1.0 mL/min. Each sample was analysed by triplicate.

Electron impact mode (EI) at 70 eV, was used for mass spectrometry analysis. The mass range varied from 35 to 300 u. Identification of volatile components in hyssop essential oil was carried out using the NIST/ADAMS library data of the GC/MS system [17] with their characteristic *m/z* values. Quantification was carried out according to the area percentage using the scan mode.

### 3.4. Statistical Analysis

SPSS Statistics 17.0 Software (Chicago, IL, USA) was used to evaluate one-way analysis of variance (ANOVA) at *p* ≤ 0.05. Canonical Discriminant Analysis was also used to establish differences between samples, and to evaluate the importance of different variables on discrimination.

## 4. Conclusions

The data obtained for each batch reveal volatile composition differences in relation to the quality of essential oil in function of the harvest period. Among all the batches analysed, the one with higher significant extraction yield belong to an irrigated plot (batch 7). In this regard, the irrigation and harvesting at the end of the maturity period are shown as the most favourable for the hyssop cultivation in Castilla-La Mancha (Spain). Most compounds with values initially outside the ranges established by the ISO9841:2007 (E) (*iso*-pinocamphone, *α*-pinene, *β*-pinene, *β*-caryophyllene, *etc*.), evolved favourably towards meeting the standard. 
